# Efficacy and Safety of Inhalation Sedation during Office Probing for Congenital Nasolacrimal Duct Obstruction

**DOI:** 10.3390/jcm10081800

**Published:** 2021-04-20

**Authors:** Chunghyun Lee, Su-Min Jeong, Gye Jung Kim, Eun-Young Joo, Myung Hee Song, Ho-Seok Sa

**Affiliations:** 1 Department of Ophthalmology, Gangneung Asan Hospital, Gangneung 25440, Korea; chlee.oph@gmail.com; 2 Department of Family Medicine, Samsung Medical Center, Seoul 06351, Korea; smjeong.fm@gmail.com; 3 Department of Ophthalmology, Asan Medical Center, University of Ulsan College of Medicine, Seoul 05505, Korea; comether83@gmail.com; 4 Department of Anesthesiology and Pain Medicine, Asan Medical Center, University of Ulsan College of Medicine, Seoul 05505, Korea; naeng82@gmail.com (E.-Y.J.); mhsong@amc.seoul.kr (M.H.S.)

**Keywords:** congenital nasolacrimal duct obstruction, epiphora, inhalation sedation, probing procedure

## Abstract

We compared the effectiveness of inhaled sevoflurane versus physical restraint during probing in children with congenital nasolacrimal duct obstruction (CNLDO). We performed a retrospective review of children with CNLDO who underwent office probing procedures by a single surgeon under sedation or restraint. Patients’ characteristics at the time of probing, including age, sex, laterality, previous non-surgical treatment, presence of dacryocystitis, outcomes of probing, and complications were compared between the sedation and restraint groups. A multivariable logistic regression analysis was performed to investigate the prognostic factors associated with the success of probing. A subgroup analysis by 12 months of age was also conducted. The overall success rate was 88.6% in 202 eyes of 180 consecutive children (mean age, 15.1 ± 7.7 months). The sedation group had a marginally higher success rates than the restraint group (93.8% vs. 85.1%, *p* = 0.056). The success rate was not significantly different between the two groups in children aged <12 months (90.9% vs. 93.1%, *p* = 0.739), but it was significantly higher in the sedation group (94.7% vs. 77.8%. *p* = 0.006) in children aged ≥12 months. Inhalation sedation was the most potent factor associated with success (adjusted odds ratio = 5.56, 95% confidence interval = 1.33–23.13, *p* = 0.018) in children aged ≥12 months. There were no surgical or sedation-related complications intra- and postoperatively. Inhaled sevoflurane sedation resulted in more successful, controlled, painless probing, particularly in children aged ≥12 months. It represents a safe, efficient alternative to general anesthesia.

## 1. Introduction

Congenital nasolacrimal duct obstruction (CNLDO) commonly occurs in infants, and often spontaneously resolves within the first year of life [1,2]. If CNLDO persists despite conservative management such as lacrimal sac massage and/or topical antibiotics, nasolacrimal duct probing may be performed [1,2]. However, the setting of probing remains controversial, particularly as office-based probing is usually performed using topical anesthesia under physical restraint. Some previous studies suggest that probing is ideally performed under general anesthesia (GA), as physical restraint might be insufficient to ensure the safe completion of probing [3,4,5]. Lee KA et al. found that the success rate of probing was 18% lower in an office setting under restraint than in a surgical facility under GA [4]. Miller et al. also reported a higher success rate under GA compared with probing under restraint (relative risk = 1.27 to 1.36) and attributed this to the fact that office-based physical restraint offers less control and provides less cooperative conditions [5]. In addition, physical restraint during the procedure might traumatize and otherwise worsen the child’s negative experience [6]. Meanwhile, there have been concerns that GA in early childhood carries short- and long-term safety issues, requires longer procedure times, and results in higher costs [7,8].

Sedation with inhaled sevoflurane has been widely used in pediatric outpatient procedures because of its rapid induction and elimination [9,10]. We hypothesized that inhalation sedation could be useful for office probing in children with CNLDO. This study aimed to assess the effectiveness and safety of sedation with inhaled sevoflurane for nasolacrimal duct probing compared to physical restraint without sedation.

## 2. Methods

### 2.1. Subjects and Study Design

This study was conducted at the Department of Ophthalmology, Asan Medical Center, Seoul, Korea and included 202 eyes of 180 children with CNLDO who underwent the probing procedure between January 2012 and February 2020. A detailed history and physical examination were performed on each patient. Major eligibility criteria included the onset of nasolacrimal duct obstruction (NLDO) symptoms prior to the age of six months, presence of at least one sign of NLDO (epiphora, increased lacrimal lake, and/or mucous discharge in the absence of an upper respiratory infection or ocular irritation), and a positive fluorescein dye disappearance test (FDDT). Exclusion criteria included any acquired NLDO, complex CNLDO, presence of facial anomaly, prior NLDO surgery, and a short follow-up period less than three months after probing. The types of previous conservative treatment were categorized into topical antibiotics only, lacrimal massage only, or both, on the basis of the patient’s history. The presence of dacryocystitis was determined by clinical signs and regurgitation of mucopurulent discharge by pressing the lacrimal sac.

Patients were assigned to receive primary office probing as either the restraint group or the sedation group according to the date of procedure. Between January 2012 and February 2015, all consecutive patients underwent probing under physical restraint and topical anesthesia without sedation (the restraint group). Between March 2015 and February 2020, all patients underwent probing under sedation with inhaled sevoflurane (the sedation group).

### 2.2. Probing Procedures

All probing procedures were performed by a single ophthalmologist (H-S Sa) in a treatment room of the outpatient clinic at Asan Medical Center. All patients remained in their own street clothes during the procedure. Prior to inhalation sedation, patients were fasted according to the institutional guidelines: clear liquid for 2 h, breast milk for 4 h, and milk and solids for 6 h before the procedure. The anesthesiologist induced sedation by spontaneous ventilation of a mixture of oxygen and sevoflurane through a facial mask. The concentration of sevoflurane was gradually increased up to 8% during induction and maintained at approximately 4% during the procedure to achieve 1.5 to 2 times minimum alveolar concentration. The facial mask was removed temporarily during the probing procedure, which was less than one minute in most cases. If the procedure time was prolonged, inhalation sedation was additionally performed at the discretion of the anesthesiologist. The anesthesiologist and assistant continuously monitored the electrocardiogram, automated blood pressure, pulse oximetry, and end-tidal carbon dioxide during the procedure. Intravenous catheters and endotracheal intubation were not required (Supplementary Video S1).

The probing procedure was performed in the same way for both groups (Supplementary Video S1). Topical anesthetic drops were placed in the affected eye. After dilation of the lower punctum with a fine punctal dilator, a Bowman probe of size #1 was inserted perpendicular to the lower eyelid margin and advanced into the ampulla. The probe was then rotated horizontally into the lower canaliculus and advanced toward the lacrimal sac while lateral traction was applied to the eyelid. When a hard stop was felt, the probe was rotated 90 degrees and advanced downward into the nasolacrimal duct until it passed through the obstructed site. The obstruction was maximally dilated by repeatedly passing and rotating the probe at the site of obstruction. Patency of the nasolacrimal duct was checked by syringing a small amount of normal saline less than 0.5 cc. In all bilateral cases, probing was performed in the right eye first, then in the left. Antibiotic (0.3% tobramycin) and steroid (0.1% fluorometholone) eyedrops were administered three times a day for two weeks postoperatively in all cases.

### 2.3. Follow-Up and Postoperative Evaluation

The patients were followed up at one and three months postoperatively. Success was defined as the complete remission of NLDO symptoms, normal height of lacrimal lake, negative FDDT, and no need for an additional surgery at three months after the primary probing.

### 2.4. Statistical Analysis

All statistical analyses were carried out using Stata version 14.1 (Stata Corp, College Station, TX, USA). We used the t-test and Chi-square test for continuous and categorical variables to examine the differences between the sedation and restraint groups. The success rate (%) was calculated as the number of successful cases divided by the total number of cases, expressed as a percentage. Logistic regression was performed to evaluate factors associated with successful outcomes. In a multivariable logistic regression, model 1 was adjusted for age in months and sex in consideration of the adjustment in previous studies and the marginally significant differences between the sedation and the restraint groups in our study. Model 2 was additionally adjusted for bilateral or unilateral CNLDO, types of previous treatments, and the presence of dacryocystitis. Subgroup analyses at the cut-off ages of 12, 18, and 24 months old were conducted.

## 3. Results

The study enrolled 202 eyes of 180 patients, and the mean age at the time of probing was 15.1 ± 7.7 months (range, 5 to 49 months). The sedation group included 81 eyes (70 patients) and the restraint group included 121 eyes (110 patients) (Table 1). The sedation group had a higher average age than the restraint group (mean months, 16.9 ± 8.5 vs. 13.6 ± 6.7, *p* = 0.004). In total, 11 cases in each group underwent bilateral probing procedures (*p* = 0.254). The mean sedation time was 9.3 ± 3.1 min in the sedation group. There were no patients in both groups who had incomplete probing due to severe obstruction. The overall success rate of probing was 88.6% (179/202 eyes). The sedation group had a marginally higher success rate than the restraint group (93.8% vs. 85.1%, *p* = 0.056). No surgical or sedation-related complications were reported intra- and postoperatively.

Table 2 shows the success rates by age cut-offs. At the cut-off age of 12 months, the success rate was not significantly different between the sedation and restraint groups in children aged <12 months (90.9% vs. 93.1%, *p* = 0.739), but it was significantly higher in the sedation group than the restraint group (94.7% vs. 77.8%. *p* = 0.006) in children aged ≥12 months. At the other cut-off ages of 18 or 24 months, the success rates tended to be higher in the sedation group compared to the restraint group, but the differences were not statistically significant (Table 2). Of the 22 patients who had bilateral probing, all 11 children in the sedation group showed successful outcomes, but 3 out of 11 children (four eyes) in the restraint group failed to achieve complete resolution after the primary probing and underwent additional procedures. One patient, for whom both eyes failed to be cleared, underwent a silicone intubation to achieve resolution. The two patients with failure in the left eye improved after a second office probing.

The factors associated with success are presented in Table 3. Overall, inhalation sedation was marginally associated with success (adjusted odds ratio (aOR) = 2.88, 95% confidence interval (CI) = 0.96–8.63, *p* = 0.058). In a subgroup aged <12 months, increasing age by months was significantly associated with success (aOR = 2.92 per 1-month increase, 95% CI = 1.18–7.24, *p* = 0.020). In a subgroup aged ≥12 months, inhalation sedation was the most potent factor associated with success (aOR = 5.56, 95% CI = 1.33–23.13, *p* = 0.018).

## 4. Discussion

Several previous studies have suggested that the success rate of probing for CNLDO could differ depending on surgical setting, the patient’s age, and the surgeon’s skill and experience [3,4,5,11,12]. We found an 88.6% overall success rate, which was comparable to those (72% to 98.1%) reported by other large case series that included probing under physical restraint or GA [4,5,11,12,13,14,15]. The success rate of the sedation group (93.8%) in this study was marginally higher than that of the restraint group (85.1%), and it also appeared higher than the success rates (72% to 86%) of office probing reported by previous studies [5,16,17]. Miller et al. performed office probing on 384 eyes of 304 children aged 6 to 15 months under restraint and topical anesthesia and reported a 75% success rate [5]. They reported a significantly lower success rate for bilateral probing (63%) than unilateral probing (80%) in an office setting, attributing this to the increased time required to probe both eyes which, when the child is conscious, increases the difficulty involved in controlling the child’s movement under physical restraint. This leads to a less controlled procedure, especially when probing the second eye [5]. In our study, three children (four eyes) failed to receive successful treatment for their bilateral cases in the restraint group, and probing of the second eye failed in all three children. This could not be verified as statistically significant, however, because of the small sample size. By contrast, there were no cases of failed bilateral probing in the sedation group. Our findings therefore appear to support the hypothesis suggested by Miller et al. [5].

In this study, the success rate of primary probing was no different between the sedation (90.9%) and restraint (93.1%) groups in children aged <12 months. However, in children aged ≥12 months, a significantly higher success rate was observed in the sedation group (94.9%), compared with the restraint group (77.8%). Interestingly, inhalation sedation was the most potent factor associated with success (aOR = 5.56) in children aged ≥12 months from the multivariable regression analysis. Another potential prognostic factor in the same age subgroup was the presence of dacryocystitis (aOR = 0.09), which was marginally associated with failure (*p* = 0.054). Lee KA et al. suggested that safely restraining older children is challenging and GA or other sedation approaches comparable to GA should be considered [3]. This is in agreement with our observation that sedation was associated with success in older children. Similarly, a large, prospective, multicenter study reported that office probing under restraint had a lower success rate than under GA (adjusted relative risk = 0.88), and they also showed no decline in success rates with increasing ages up to the age of 36 months [4]. In our study, however, we evaluated other cut-off ages of 18 and 24 months, but we were not able to verify significant differences between the two groups because of the small sample size of older children. Further study with a larger cohort would allow us to make a more valid comparison.

The success rate of the sedation group (93.8%) in our study was comparable to those of previous studies under GA (84% to 92%) [11,13,16]. Clark RA reported a 92% success rate (66/72 eyes) of probing under GA [13]. Parveen et al. also reported a high success rate (84% to 94%) of probing under GA in children aged 6 to 18 months, suggesting probing under GA is a safe option to reduce the potential risk of intraoperative damage to the lacrimal system [16]. Despite the advantage of GA offering successful probing, it also has disadvantages such as short- and long-term safety concerns, a longer procedure time, and higher costs [7,8]. As an alternative to GA, Movaghar et al. examined the use of intravenous propofol sedation for probing in 22 children aged 11.5 to 39 months and reported an 84% success rate, comparable to those of previous studies under GA, with shorter procedure (10.5 min) and recovery (13.5 min) times and better cost-effectiveness than GA [8]. However, intravenous propofol sedation has the disadvantage of requiring intravenous line placement in conscious children, compared with the inhalation sedation which does not.

The aim of sedation during probing in children is to achieve immobility and to minimize physical discomfort, pain and potential psychological trauma which may arise from the use of physical restraint. Sevoflurane is a well-established safe anesthetic agent used for more than 20 years, and it is suitable for achieving immobility mainly by its analgesic effect [18]. Its low blood/gas partition coefficient (0.69) results in a more rapid uptake and fast elimination [18]. In addition to the pharmacokinetic property of sevoflurane, an agreeable odor and lack of pungency provides an obvious advantage in pediatric anesthesia [19]. Compared to desflurane and isoflurane, sevoflurane has been described as the agent of choice for mask induction in children due to its lack of airway irritation [20]. The common adverse events during recovery from sevoflurane sedation include nausea/vomiting, coughing, and agitation/delirium [18,19], but there were no significant adverse events reported intra- and postoperatively in our study. There might be safety concerns about not placing an IV line, but our results show that sevoflurane sedation for quick minor procedures is relatively safe. Sato et al. reported that only sevoflurane provided efficient sedation and adequate analgesia for laser photocoagulation treatment of retinopathy in prematurity compared with other intravenous sedative and analgesic agents and local anesthetics [21].

For quick minor procedures such as probing for CNLDO, sedation with inhaled sevoflurane would be a better alternative than GA, without losing the advantages of GA. In their study on parental satisfaction with probing in 81 children, Goldblum et al. reported that most caregivers would prefer office probing (81%) to facility probing under GA (13%; 6% were unsure) [22]. This preference was mainly due to the longer procedure time, higher costs, and potential risks and stress associated with GA. Nevertheless, caregivers were also concerned about potential psychological trauma from restraint without sedation [22]. For children, it may be a frightening experience to change into surgical gowns and enter the operating room. In our study, the mean sedation time was 9.3 min and all children remained in their own street clothes during the procedure. Although we could not compare inhalation sedation and GA with respect to the time required, it seems obvious that inhalation sedation can save an enormous amount of time for the procedure. Regarding medical costs charged at our institution, the cost for probing under inhaled sedation was more expensive than that for probing under restraint (approximately, 415 USD vs. 150 USD), but both were less than GA (1186 USD). We did not conduct a quantitative analysis to compare parental satisfaction between the two groups because of a lack of information. However, no caregivers refused to allow inhalation sedation when they were informed, and most were satisfied with the procedure conducted under sedation.

There are a number of limitations to the findings in this study. First, the sedation group was older on average than the restraint group, mainly because of the different time periods of procedures between the two groups. We assume that an increasing number of older children were referred to our clinic, a tertiary referral hospital, for probing after the sedation procedure room was set up. Given the negative prognostic effect of increasing age on successful probing, the age difference between the two groups does not seem to undermine the usefulness of sedation identified in this study. Second, we have a room equipped for inhalation sedation and monitoring in which to perform office probing. This may not be readily set up in other hospital environments. However, we believe that the sedation room offers a more convenient, effective, and satisfying environment for caregivers and children, compared with the operating room. Third, we could not validate other prognostic factors such as bilateral CNLDO, severe symptoms, canalicular stenosis, and obstruction types mainly because of the small sample size and lack of information. A further prospective study with a larger cohort is required.

## 5. Conclusions

In conclusion, this study demonstrated that nasolacrimal duct probing for CNLDO had higher success rates when performed under sedation with inhaled sevoflurane than under physical restraint without sedation, especially in children aged ≥12 months. In a subgroup aged ≥12 months, inhalation sedation was the most significant factor associated with success of probing. Therefore, we believe that sedation with inhaled sevoflurane in an office setting is safe, with no significant complications reported, and also efficient, without the need for an intravenous catheter or endotracheal intubation during probing in children with CNLDO.

## Figures and Tables

**Table 1:** Comparison of patients’ characteristics and outcomes for office probings under sedation and restraint.

	Group		
Characteristics	Sedation	Restraint	*p* Value
No. patients (no. eyes)	70 (81)	110 (121)	
Age, months	16.9 ± 8.5	13.6 ± 6.7	0.004
≥12 months, *n* (%)	51 (72.9)	58 (52.7)	0.007
Male, *n* (%)	44 (62.9)	55 (50.0)	0.091
Bilateral, *n* (%)	11 (15.7)	11 (10.0)	0.254
Dacryocystitis, eyes (%)	3 (3.7)	9 (7.4)	0.271
Previous treatment, eyes (%)			0.325
Lacrimal massage and topical antibiotics	49 (60.5)	70 (57.9)	
Topical antibiotics only	11 (13.6)	12 (9.9)	
Lacrimal massage only	0 (0)	2 (1.7)	
None	21 (25.9)	37 (30.6)	
Follow-up, months	9.8 ± 4.9	13.7 ± 12.8	0.001
Success rates, eyes (%)	76/81 (93.8)	103/121 (85.1)	0.056

**Table 2:** Success rates of office probings by age of months.

Age	Success Rate, *n* (%)			
	Total (*n*, 202 Eyes)	Sedation (*n*, 81 Eyes)	Restraint (*n*, 121 Eyes)	*p* Value
<12 months	74/80 (92.5)	20/22 (90.9)	54/58 (93.1)	0.739
≥12 months	105/122 (86.1)	56/59 (94.9)	49/63 (77.8)	0.006
<18 months	138/156 (88.5)	51/54 (94.4)	87/102 (85.3)	0.089
≥18 months	41/46 (89.1)	25/27 (92.6)	16/19 (84.2)	0.368
<24 months	156/175 (89.1)	62/66 (93.9)	94/109 (86.2)	0.112
≥24 months	23/27 (85.2)	14/15 (93.3)	9/12 (75.0)	0.183
Total	179/202 (88.6)	76/81 (93.8)	103/121 (85.1)	0.056

**Table 3:** Logistic regression analysis evaluating the factors associated with success of probing.

	Univariate Analysis		Multivariate Analysis			
	Crude OR (95% CI)	*p* Value	aOR (95% CI)	*p* Value	aOR (95% CI)	*p* Value
Total (Number of Eyes = 202)			Model 1		Model 2	
Inhalation sedation	2.66 (0.94–7.47)	0.064	3.01 (1.03–8.74)	0.043	2.88 (0.96–8.63)	0.058
Age ≥12 months	0.51 (0.19–1.36)	0.180	0.42 (0.15–1.14)	0.090	0.36 (0.12–1.03)	0.058
Sex, female	0.68 (0.28–1.61)	0.378	0.79 (0.32–1.95)	0.614	0.91 (0.35–2.35)	0.842
Bilateral	1.37 (0.44–4.25)	0.589			0.98 (0.29–3.32)	0.973
Lacrimal massage and topical antibiotics (vs. none)	2.55 (1.01–6.42)	0.047			2.41 (0.91–6.37)	0.075
Dacryocystitis	0.35 (0.09–1.41)	0.141			0.29 (0.06–1.36)	0.117
Age <12 months (number of eyes = 79)						
Inhalation anesthesia	0.75 (0.13–4.45)	0.756	0.10 (0.01–1.49)	0.094	0.12 (0.01–1.77)	0.122
Months (per 1-month increase)	2.04 (1.22–3.41)	0.007	3.34 (1.44–7.71)	0.005	2.92 (1.18–7.24)	0.020
Sex, female	0.78 (0.15–4.13)	0.771	0.23 (0.02–2.18)	0.202	0.24 (0.02–2.75)	0.253
Bilateral	NA	NA			NA	NA
Lacrimal massage and topical antibiotics (vs. none)	4.50 (0.76–26.6)	0.097			2.81 (0.30–26.20)	0.364
Dacryocystitis	0.53 (0.05–5.21)	0.586			2.57 (0.09–75.19)	0.583
Age ≥12 months (number of eyes = 123)						
Inhalation anesthesia	5.23 (1.42–19.25)	0.013	4.89 (1.29–18.57)	0.020	5.56 (1.33–23.13)	0.018
Months (per 1-month increase)	1.02 (0.95–1.10)	0.503	1.02 (0.94–1.09)	0.680	1.02 (0.94–1.10)	0.683
Sex, female	0.63 (0.23–1.76)	0.380	0.84 (0.28–2.51)	0.756	1.00 (0.30–3.36)	1.000
Bilateral	0.95 (0.28–3.19)	0.935			0.61 (0.15–2.42)	0.482
Lacrimal massage and topical antibiotics (vs. none)	2.07 (0.68–6.29)	0.198			2.02 (0.58–6.99)	0.268
Dacryocystitis	0.14 (0.02–1.10)	0.062			0.09 (0.01–1.04)	0.054

CI = confidence interval; aOR = adjusted odds ratio, NA = not applicable, no reintervention cases in both eyes (<12 months); Model 1 was adjusted for age (months) and sex; Model 2 was adjusted for age (months), sex, bilateral, previous treatment, and dacryocystitis.

## Data Availability

The data that support the findings of this study are available from the corresponding author, upon reasonable request.

## Supplementary Materials

The following are available online at https://www.mdpi.com/article/10.3390/jcm10081800/s1, Supplementary Video S1. Video showing the probing procedure for congenital nasolacrimal duct obstruction under sedation with inhaled sevoflurane.
